# Effect of Irradiation Temperature and Atmosphere on Aging of Epoxy Resins for Superconducting Magnets

**DOI:** 10.3390/polym16030407

**Published:** 2024-02-01

**Authors:** David Mate Parragh, Christian Scheuerlein, Noémie Martin, Roland Piccin, Federico Ravotti, Giuseppe Pezzullo, Torsten Koettig, Dirk Lellinger

**Affiliations:** 1 European Organization for Nuclear Research (CERN), 1211 Meyrin, Switzerland; 2 Chemistry Department, Claude Bernard University Lyon 1, 69100 Villeurbanne, France; 3 Fraunhofer Institute for Structural Durability and System Reliability, 64289 Darmstadt, Germany; dirk.lellinger@lbf.fraunhofer.de

**Keywords:** accelerator magnets, FCC, HL-LHC, epoxy resin, irradiation, DMA

## Abstract

The superconducting magnets of future particle accelerators will be exposed to high irradiation doses at cryogenic temperatures. To investigate the effect of irradiation temperature and atmosphere on the aging behavior, we have characterized the changes in thermomechanical properties of six epoxy resins for potential use in superconducting magnets after irradiation up to 20 MGy in ambient air, inert gas, and liquid helium. Based on the results obtained by Dynamic Mechanical Analysis (DMA), we discuss the effect of irradiation temperature and the presence of oxygen. The irradiation temperature can have a strong influence on the rates at which cross-linking and chain scission occur.

## 1. Introduction

Polymers are needed in superconducting magnets for dielectric insulation [1] and for the impregnation of magnet coils made of brittle conductors like Nb_3_Sn [2]. In future particle accelerators like the Future Circular Collider (FCC) project [3,4], the magnets will be exposed to increasingly high radiation doses. As an example, the predicted peak dose in the HL-LHC [5] inner triplet coils is 30 MGy [6].

Epoxy resins are thermosetting polymers with good dielectric and mechanical properties that are commonly used for the impregnation of large coils for magnets, for coil windings for motors and generators, and as the matrix material of fiber-reinforced composites. Radiation damage of such epoxy resins has been extensively studied [7].

Previously, we have described the aging of different epoxy resin systems for potential use in superconducting magnets during irradiation in ambient air [8]. Since the polymers in superconducting magnets are irradiated at cryogenic temperatures in the absence of oxygen, in the present study we investigate the effect of the irradiation temperature and atmosphere. For this purpose, we have irradiated the same epoxy resins in three different environments: at 20 °C, either in ambient air or inert gas, and immersed in liquid helium at 4.2 K.

To assess aging processes and determine aging rates, we apply Dynamical Mechanical Analysis (DMA). DMA storage and loss moduli evolutions reveal the effect of the competing influence of cross-linking and chain scission on the glass transition temperature (*T_g_*) and on the molecular weight between cross-links of the macromolecules. The irradiation environment, and in particular the irradiation temperature, can substantially impact the irradiation-induced aging of epoxy resins.

## 2. Materials and Methods

### 2.1. The Samples

In the present study, six epoxy resin systems that can be used for the vacuum impregnation [9] of coils for magnets and rotating machines have been characterized before and after irradiation with different sources to dose levels up to 20 MGy.

#### 2.1.1. CTD101K

The CTD101K epoxy system [10] is the baseline impregnation system for the Nb-Ti MCBXF and Nb_3_Sn MQXF HL-LHC superconducting magnets [11,12,13]. This epoxy system consists of diglycidyl ether of bisphenol-A (DGEBA), a carboxylic anhydride hardener, and an accelerator. These components are mixed in the proportion CTD101K resin:hardener:accelerator = 100 parts by weight (pbw): 90 pbw:1.5 pbw. The curing temperature cycle comprises two plateaus: 5 h–110 °C and 16 h–125 °C post-curing.

#### 2.1.2. MSUT

The so-called MSUT Twente epoxy system consists of the Araldite MY740 bisphenol A/epichlorohydrin resin (type DGEBA, Mw < 700 g/mol), cured with the carboxylic anhydride hardener of mixed composition Aradur HY906, and the amine accelerator DY062 in the respective ratio 100 pbw:90 pbw:0.2 pbw. The curing temperature cycle comprises two plateaus: 4 h–85 °C and 16 h–110 °C post-curing.

#### 2.1.3. MY750

The epoxy resin system MY750 is composed of the Araldite MY750 bisphenol A/epichlorohydrin resin, type DGEBA, Mw < 700 g/mol (100 pbw), and the aliphatic polyamine hardener Aradur HY5922 (55 pbw) from Huntsman Corporation. The curing temperature cycle comprises two plateaus: 6 h–40 °C and 3 h–80 °C post-curing.

#### 2.1.4. Mix61

The so-called Mix61 epoxy system [14,15] is composed of a diglycidyl ether of bisphenol-A (DGEBA) resin, an aromatic hardener of the amine type, a high molecular weight co-reactant of the amine type, and a liquid low molecular weight additive. The curing temperature cycle comprises two plateaus 16 h–60 °C and 24 h–100 °C post curing.

More details about the CTD101K, MY750, Mix61, and MSUT processing can be found in reference [16].

#### 2.1.5. CEA Mix

The two-component resin system Huntsman Araldite^®^ CY192-1, a cycloaliphatic epoxy resin (100 pbw), and its corresponding anhydride-type hardener, Huntsman Aradur^®^ HY 918-1 (100 pbw) [17], have been used by CEA Saclay for the impregnation of Nb_3_Sn quadrupole coils [18]. The applied curing cycle recommended by CEA Saclay comprises three isothermal plateaus at 80 °C–24 h, 120 °C–34 h and at 130 °C–12 h.

#### 2.1.6. Araldite F

The epoxy system referred to as Araldite F is used by the company ASG Superconductors S.p.A., Genoa, Italy, for impregnation of magnet coils and consists of the bisphenol A/epichlorohydrin resin (type DGEBA) Araldite F, the carboxylic anhydride hardener Aradur HY 905, and the polyglycol flexibilizer DY 040. The resin, hardener, and flexibilizer are combined in the ratio of 100 pbw:100 pbw:10 pbw, respectively. The Araldite F/Aradur HY 905/flexibilizer DY 040 system does not contain the accelerator DY 061, and filler that is recommended by Huntsman [19]. The applied curing cycle comprises two plateaus 100 °C–10h and 135 °C–48 h.

The samples were cut from 3 mm or 4 mm thick pure epoxy resin plates that were produced by vacuum impregnation at the CERN polymer lab. All samples of a given material were cut from the same plate, thus eliminating uncertainties related to the sample production processes.

Sample curing inside the mold was achieved in a forced convection furnace. The stated curing temperatures are nominal furnace temperature values, and the estimated uncertainty of the plate temperature during the isothermal plateaus is ±2 °C. During heating, the nominal temperature ramp was 10 °C per hour. Sample cooling occurred by natural convection after switching off the furnace. Unlike in large coils, where temperature gradients during curing across the coil are often unavoidable, in the epoxy sample plates, temperature gradients across the plate inside its mold are negligible.

### 2.2. Radiation Sources

#### 2.2.1. Gamma Irradiation

^60^Co irradiation has been performed at the Gammatec facility at the Marcoule site of the company Synergy Health Marseille SAS, Marseille, France (a Steris company) with a dose rate of about 2 kGy/h in ambient air at a temperature of 20–25 °C. During irradiation, the sample holders were continuously rotated for better dose homogeneity. Dosimetry measurements were performed with Perspex dosimeters, which were attached at different positions inside and outside the sample holders.

#### 2.2.2. Proton Irradiation

Irradiations with 24 GeV protons have been performed at the CERN IRRAD facility with a proton fluence of about ~1.434 × 10^16^ p/cm^2^/week [20]. More details about the IRRAD facility and the sample dosimetry measurements can be found in [8].

**Figure 1:**
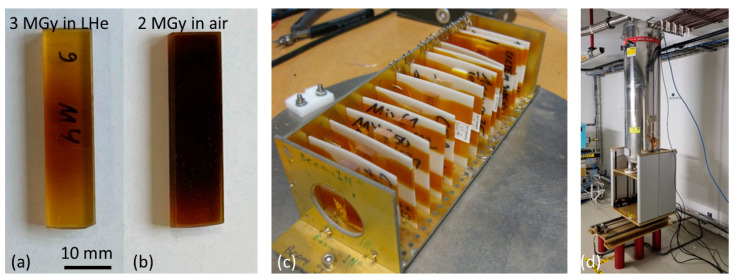
MY750 samples after 24 GeV proton irradiation (**a**) to 3 MGy immersed in liquid helium and (**b**) to 2 MGy behind the cryostat in ambient air. (**c**) Sample holder with 13 samples for insertion in the LHe cryostat, and (**d**) cryostat inside the IRRAD facility.

The irradiations at IRRAD have been performed in different environments and at different temperatures, notably in air at ambient temperature, in inert gas at ambient temperature, and immersed in liquid helium (LHe) at 4.2 K. Samples, sample holders, and cryostats used for the irradiations in LHE are presented in Figure 1. The proton beam passes through the cryostat and the samples. For dose determination, aluminum witness samples are placed in front and behind the samples, and the accumulated proton dose is determined with the activation-foil technique, as detailed in [21].

The effect of the irradiation environment is obvious from the comparison of the visual aspects of the initially identical MY750 samples after irradiation inside the cryostat in LHe (Figure 1a) and behind the cryostat in ambient air (Figure 1b).

### 2.3. Dynamic Mechanical Analysis (DMA)

Irradiation-induced changes in the properties of viscoelastic materials were monitored by DMA. The storage modulus (G′), which is related to sample stiffness, and the loss modulus (G″), which is a measure of the energy dissipated during the applied torsion oscillation, were recorded during temperature sweeps with an Anton Paar, Graz, Austria, Dynamical Mechanical Analyser MCR702e. The thermal expansion of the sample was estimated from the temperature gap change between the sample fixtures during the temperature sweep.

Measurements using solid bars with dimensions of 4 mm × 10 mm × 40 mm were performed according to ASTM D4065, DIN EN ISO 11357, using rectangular fixtures at a frequency of 1 Hz and a temperature ramp of 2 K/min. The glass transition temperature (*T_g_*) was determined by three methods: (a) the G′(T) onset according to ISO 11357; (b) the G″(T) maximum according to ASTM 4065; and (c) the tan δ(T) maximum. The G′(T) transition onset was determined with the Anton Paar RheoCompass 1.30 software as the intersection of two tangent lines fitted to G′(T) [22].

For polymers subjected to small strains, the molecular weight between the cross links (*M_c_*) and the cross link density can be calculated from the rubbery shear modulus (*G′_rubbery_*) above *T_g_*, the universal gas constant R = 8.314 J/mol·K, the absolute temperature (*T*), and the polymer density (ρ) [23,24,25].

The CTD101K and MY750 master curves have been achieved with an ARES rheometer with nitrogen cooling. Shear moduli have been acquired in the frequency range from 0.02 Hz to 30 Hz with temperature steps of 5 °C in the temperature range from −120 °C to 90 °C, of 4 °C up to 110 °C, and of 2 °C above 122 °C. The temperature steps were varied to guarantee an overlap of the y-ranges of the different DMA spectra, which is required for master curve construction.

From the measured DMA spectra at different temperatures, master curves were constructed using reference temperatures of −100 °C and 40 °C, respectively. For that, an iterative procedure was used. The x-axis of the *G′*(*f*) curve measured at the reference temperature *T_ref_* was not modified. Then, the x-axis of the next curve is multiplied with a so-called shift factor *a_T_* so that the first curve and this curve form a contiguous curve, which is modeled by a smoothing cubic spline. The shift factor *a_T_* is varied so that the sum of squared deviations between the smoothing cubic spline and the shifted curve is minimized. This procedure is then repeated for all other *G′*(*f*) spectra. After all curves are included in the smoothing cubic spline, some additional iterations are used for the fine-adjustment of the shift factors *a_T_*.

### 2.4. Thermal Expansion Measurement with the DMA Instrument

During DMA temperature sweeps, the distance between the sample clamps at a constant tensile pre-load of 0.5 N is recorded. The temperature-induced sample length change causes a corresponding change in the distance between the sample clamps. This can be exploited to determine the thermal expansion coefficients of the sample materials, as it was previously reported for a DMA instrument operating in tension mode [26].

In the following, we refer to changes in the distance between the sample clamps as a gap change. Using reference materials with known thermal expansion, we have derived a correction function with which linear thermal expansion coefficients can be calculated from the gap change. For 40 mm long samples at a temperature ramp rate of 2 K/min, the linear expansion coefficient α (×10^−6^ K^−1^) is calculated from the relative gap change in percent according to Formula (1):

α (×10^−6^ K^−1^) = 0.7172 × relative gap change (%) + 17.99 (1)

## 3. Results

### 3.1. Glass Transition and Thermal Expansion of the Non-Irradiated Epoxy Resins

**Figure 2:**
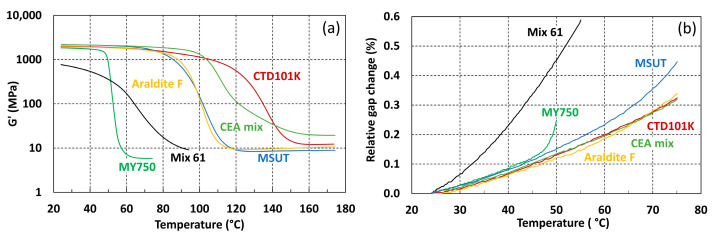
Comparison of (**a**) the storage modulus G′ (T) and (**b**) thermal expansion evolutions of the non-irradiated epoxy resins of the present study.

The glass transition onset of the six non-irradiated epoxy resins varies over a wide range from below room temperature (RT) to about 120 °C, as can be seen in the storage modulus evolutions recorded during the DMA temperature sweeps shown in Figure 2a. In Figure 2b, the thermal expansion of the different epoxy resins with respect to the reference temperature of 25 °C is shown. Below *T_g_*, the storage moduli and thermal expansion coefficients of the isotropic unfiled epoxy systems are similar. A strong increase in the thermal expansion coefficient indicates the MY750 glass transition. The glass transition onset of the Mix61 resin system is at −38 °C and is not visible in Figure 2.

The glass transition temperatures and linear thermal expansion coefficients in the temperature range 30–45 °C of the non-irradiated epoxy resin systems are summarized in Table 1. The estimated uncertainty of the *T_g_* values is smaller than ±2 °C. The CTD101K system has the highest *T_g_*. The thermal expansion coefficients of the unfilled epoxy systems are similar except for Mix61, whose *T_g_* is below RT. The estimated uncertainty of the thermal expansion coefficients is smaller than ±5%.

### 3.2. Low Temperature DMA and Master Curves of Unirradiated CTD101K and MY750

**Figure 3:**
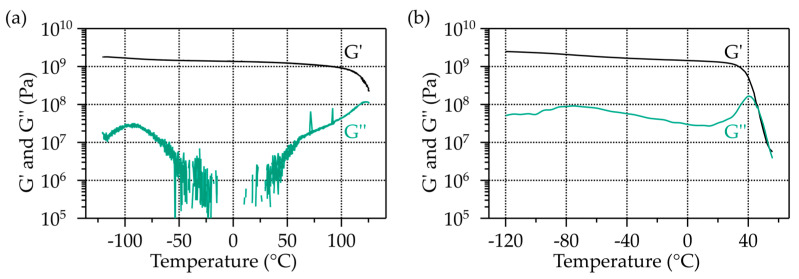
G′(T) and G″(T) of (**a**) CTD101K and (**b**) MY750 measured with a frequency of 1 Hz.

Master curves have been established for the two epoxy resin systems, MY750 and CTD101K, which exhibit the lowest and highest tan δ max *T_g_* of the materials studied here. G′(T) of CTD101K and MY750 measured at a frequency of 1 Hz are compared in Figure 3.

The CTD101K epoxy system exhibits two relaxation regions that can be recognized by a G″ maximum, one at about −94 °C, and the main relaxation (glass transition) occurs at 122 °C. The MY750 epoxy resin has one relaxation at about −75 °C and the main relaxation at about 45 °C.

**Figure 4:**
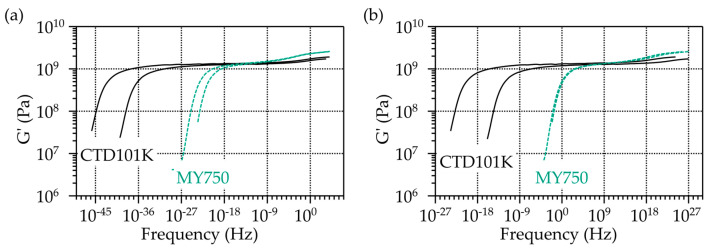
Comparison of CTD101K and MY750 master curves established from G′. Reference temperatures are (**a**) −100 °C and (**b**) 40 °C. The two curves for each material arise from measurements of two samples of the same material.

The CTD101K and MY750 master curves that have been established from a series of G′(T) measurements performed at different frequencies and temperatures are compared in Figure 4.

### 3.3. Effect of Ambient Temperature Irradiation in Air on T_g_ and G′_rubbery_

**Figure 5:**
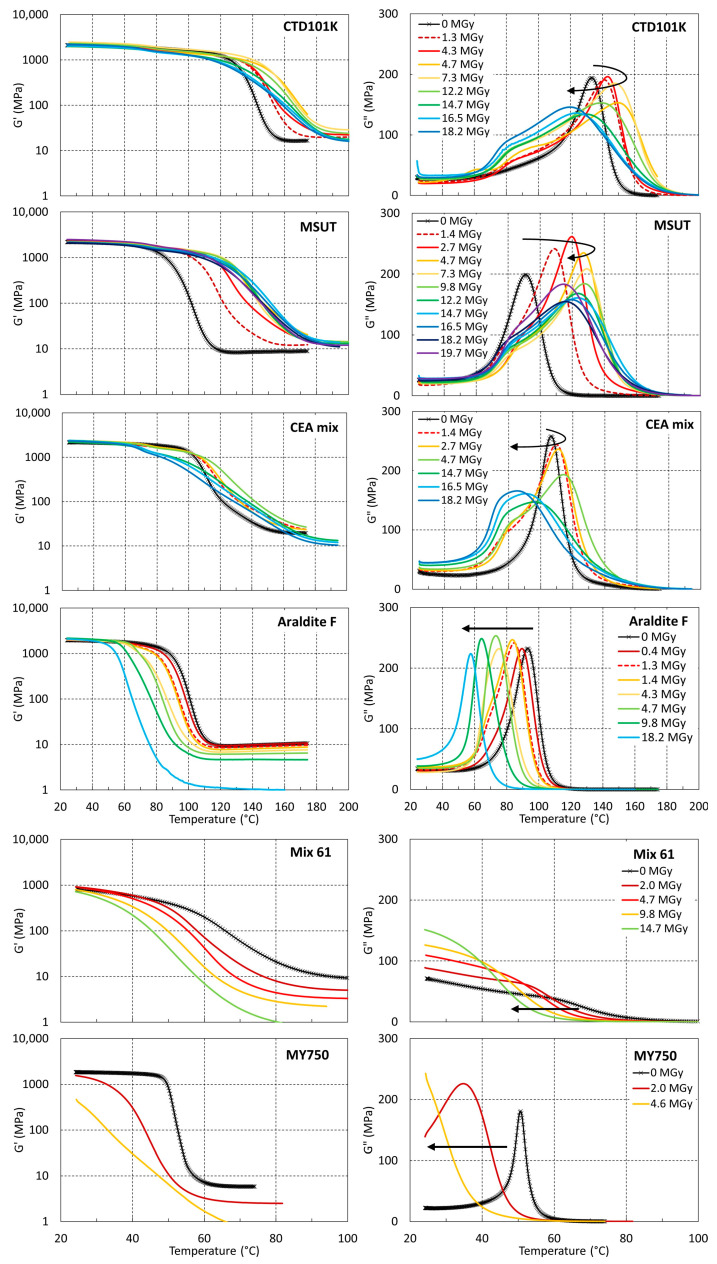
G′(T) and G″(T) evolutions measured after different proton doses absorbed in ambient air.

The G′(T) and G″(T) results of the different epoxy resin systems after ambient air proton irradiations up to 20 MGy are shown in Figure 5. For CTD101K, CEA mix, and MSUT epoxy resin systems, an increase in *T_g_* with increasing dose is initially observed, presumably due to irradiation-induced cross-linking. The maximum *T_g_* of these resins is achieved after absorption at a dose of about 5 to 8 MGy. At higher doses, *T_g_* and *G′_rubbery_* are reduced, indicating that chain scission is the dominant process.

During irradiation of the Araldite F, Mix61, and MY750 epoxy systems, a *T_g_* and *G′_rubbery_* increase is not observed, indicating that in these resins, irradiation-induced cross-linking does not occur or that its rate is small compared to the chain scission rates.

**Figure 6:**
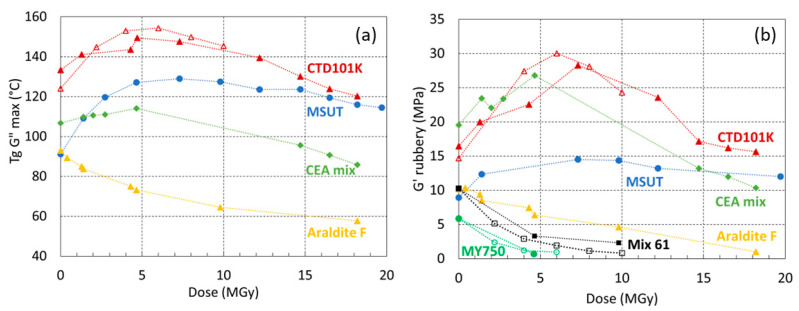
(**a**) *T_g_* G″ max and (**b**) *G′_rubbery_* as a function of absorbed dose (^60^Co gamma in open symbols or 24 GeV proton irradiation in full symbol) in ambient air. The dashed lines are a guide to the eye.

Figure 6 compares the *T_g_* and *G′_rubbery_* evolutions as a function of absorbed dose. CTD101K and MSUT maintain a comparatively high *G′_rubbery_* of up to 20 MGy. The *T_g_* and *G′_rubbery_* evolutions of MSUT exhibit the smallest irradiation-induced chain scission rate, indicating that this epoxy system has outstanding radiation resistance.

The MY750 and Mix61 epoxy systems degrade at a comparatively high rate, and these materials are not suited for applications in high-dose irradiation environments.

The effect of gamma ray and proton irradiation on the CTD101K *T_g_* evolution is compared in Figure 6a, and the effect of both radiation types on the CTD101K, Mix61, and MY750 *G′_rubbery_* evolution is compared in Figure 6b, showing that both radiation sources have a similar cross-linking and chain scission efficiency.

### 3.4. Effect of Irradiation Temperature and Atmosphere

**Figure 7:**
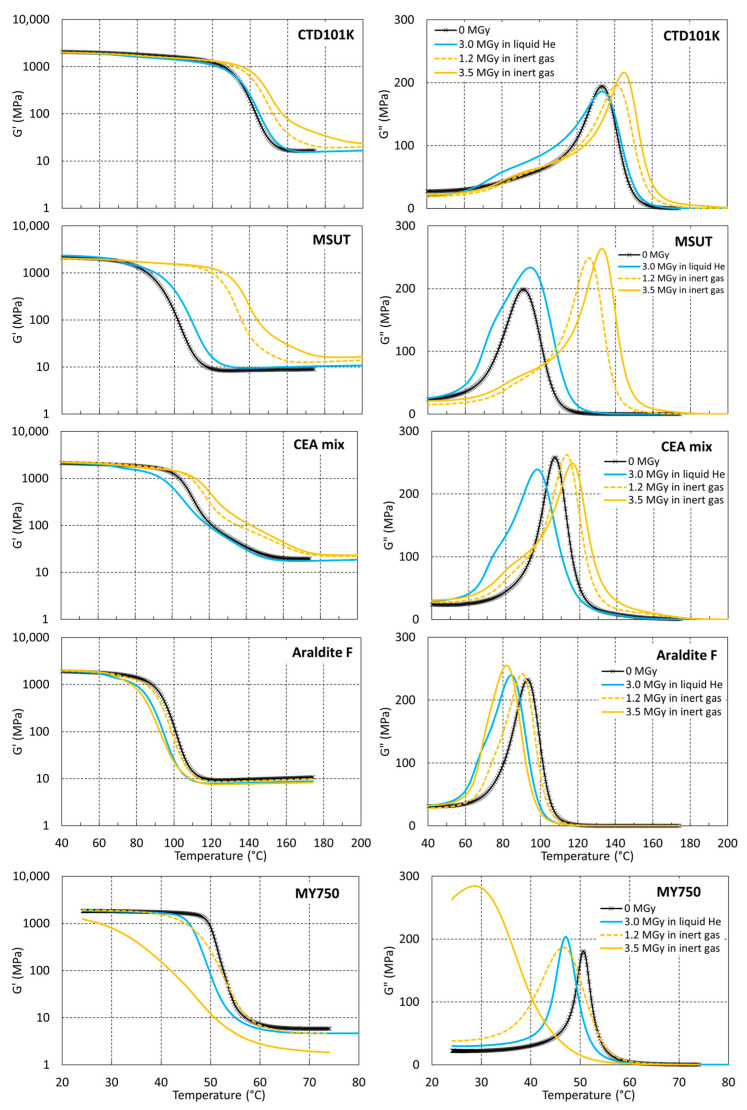
G′(T) and G″(T) of CTD101K, MSUT, CEA mix, Araldite F, and MY750 unirradiated and after proton irradiation in LHe at 4 K and inert gas at RT.

To determine the influence of the irradiation temperature on the cross-linking and chain scission rates, storage and loss moduli have been measured before and after proton irradiation of the same materials in inert gas at RT and immersed in LHe. Figure 7 compares G′(T) and G″(T) of the different epoxy systems before irradiation, after a dose of 3.0 MGy absorbed in LHe, and after doses of 1.2 MGy and 3.5 MGy absorbed in inert gas.

The effect of the irradiation temperature on the cross-linking efficiency is obvious for CTD101K, MSUT, and CEA mixes, whereas in LHe, irradiation-induced cross-linking as manifested by an increase in *T_g_* is strongly reduced (MSUT) or not observed (CTD101K and CEA mixes).

**Figure 8:**
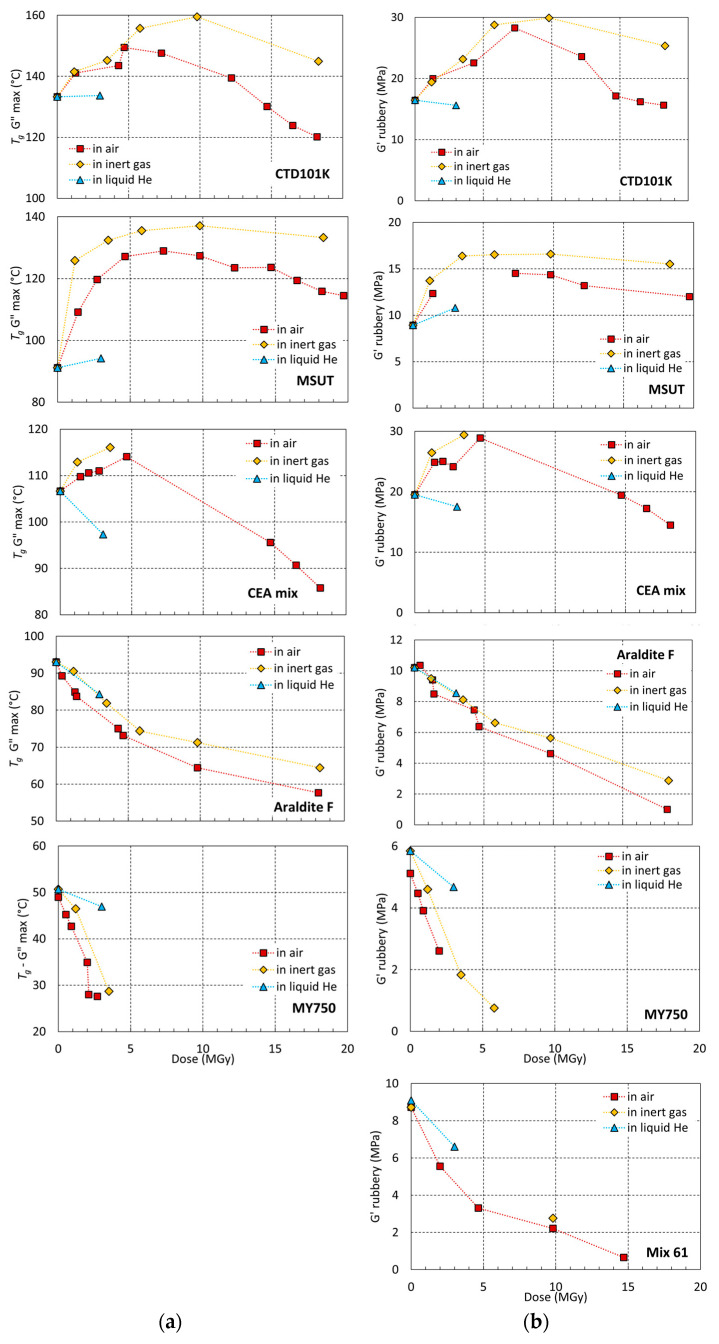
(**a**) Glass transition temperature *T_g_* G″ max and (**b**) *G′_rubbery_* of CTD101K, CEA mix, MSUT, Araldite F, MY750, and rubbery shear modulus of Mix 61 as a function of absorbed dose (^60^Co gamma or 24 GeV proton irradiation) in ambient air, at RT in inert gas, and immersion in liquid helium. The dashed lines are a guide to the eye.

In Figure 8, *T_g_* and *G′_rubbery_* of CTD101K, MSUT, CEA mix, Araldite F, MY750, and Mix61 are plotted as a function of the proton dose absorbed in different environments. The highest *T_g_* and *G′_rubbery_* values are achieved in inert gas at ambient temperature. The presence of oxygen reduces the cross-linking rate, presumably because oxygen diffuses into the polymer and reacts with radicals formed by the irradiation, thus preventing their reaction with other molecular chains.

In the epoxy resins CTD101K, MSUT, CEA mix, Mix 61, and MY750, the chain scission rate as manifested by a reduction of *T_g_* and *G′_rubbery_* is highest in ambient air, presumably because oxygen can react with irradiation-induced radicals, thus preventing the recombination of cut chains.

For all epoxy systems, except Araldite F, at liquid helium temperature, the cross-linking rate is reduced with respect to the ambient temperature cross-linking rate.

### 3.5. Comparison of T_g_ and G′_rubbery_

**Figure 9:**
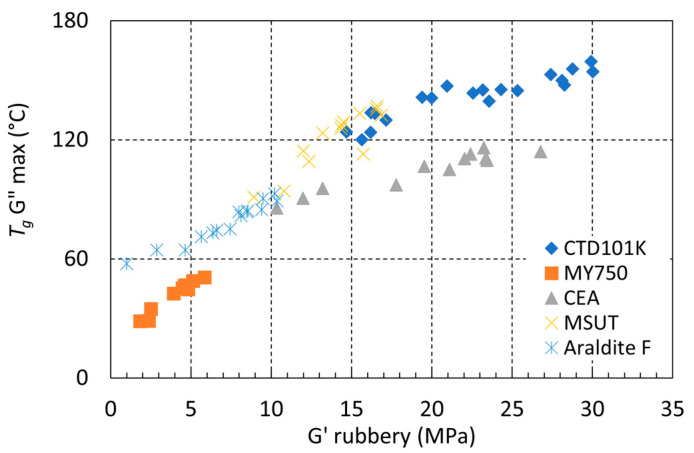
*T_g_* G″max as a function of *G′_rubbery_* of CTD101K, MSUT, MY750, Araldite F, and CEA mix before and after irradiation to different doses in different environments.

In Figure 9, the glass transition temperature *T_g_* G″ max is presented as a function of *G′_rubbery_* for all CTD101K, MSUT, CEA mix, Araldite F, and MY 750 samples before and after irradiation to different doses in different environments. Mix61 is not included because it was not possible to detect a G″ maximum for this resin.

We observe a similar *T_g_* vs. *G′_rubbery_* relation for the different epoxy resins studied. After an initial increase of *T_g_* with increasing *G′_rubbery_*, at higher *G′_rubbery_* values, a *T_g_* plateau is achieved, and further formation of cross-links only slightly changes *T_g_*.

### 3.6. Effect of Irradiation on Thermal Expansion

**Figure 10:**
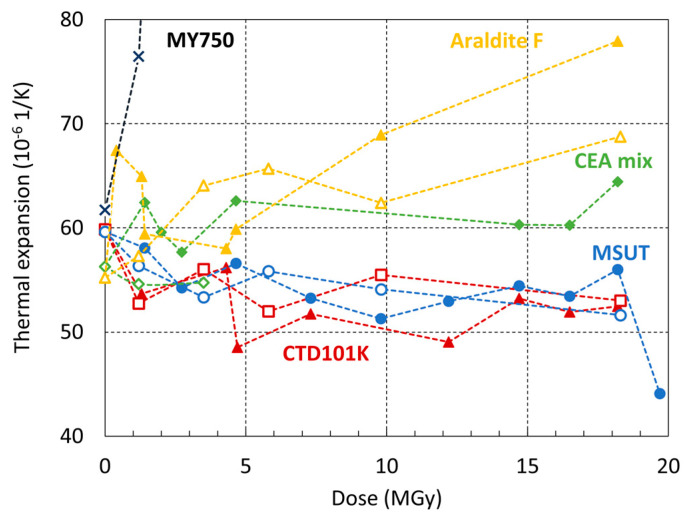
Thermal expansion in the temperature interval 30 to 45 °C as a function of the proton dose absorbed in ambient air (full symbols) and in inert gas (empty symbols).

In Figure 10, the linear thermal expansion coefficients of the different epoxy resins are plotted as a function of the dose absorbed in ambient air and in inert gas at RT. The MY750 thermal coefficient increases strongly already after absorption of low doses because its *T_g_* drops below 45 °C. Similarly, the Mix61 thermal coefficient (not shown in Figure 10) changes strongly with increasing dose.

For the other epoxy resins, changes in thermal coefficients are comparatively small and partly within experimental uncertainties (the estimated uncertainty of the thermal expansion coefficients determined during the DMA temperature sweeps is smaller than ±5%). However, for Araldite, F α seems to increase significantly after absorption of high doses, while for CTD101K and MSUT, a slight decrease of α with increasing dose is observed.

## 4. Discussion and Conclusions

### 4.1. Analytical Potential of DMA to Reveal Irradiation-Induced Aging

High-energy irradiation impacting polymer materials induces the formation of free radicals by excitation and ionization, which leads to modifications of the polymer network by simultaneous cross-linking and scission [28]. The DMA results presented here reveal changes in *T_g_* and *G′_rubbery_*, which can be used to monitor aging phenomena involving cross-linking and chain scission. For the unfilled thermosets, the average molecular weight between crosslinks can be derived by measuring the shear modulus in the rubbery plateau region. Chain scission increases the average molecular weight between cross-links, while cross-linking leads to the opposite effect.

### 4.2. Effect of the Irradiation Atmosphere

To investigate the effect of irradiation on the atmosphere, we have irradiated the same epoxy resin systems at ambient temperature in inert gas and in air. In both atmospheres, the CTD101K, MSUT, and CEA mix systems exhibit an initial increase in *T_g_* and *G′_rubbery_*, indicating that the irradiation-induced cross-linking rate is initially exceeding the chain scission rate. In these materials, a maximum *T_g_* and cross-link density are achieved after a dose of between 5 and 10 MGy is absorbed in the air. At higher doses, chain scission becomes the dominant process, and *T_g_* and *G′_rubbery_* decrease.

The cross-linking of polymers is a radical reaction that can be inhibited by the presence of oxygen due to the reaction between radicals and oxygen. This is presumably the reason why the cross-linking rate during irradiation in air is reduced with respect to the cross-linking rate during inert gas irradiation.

Cross-linking is not observed in the epoxy systems Araldite F, Mix 61, and MY750. In these resins, the chain scission rate, as manifested by a reduction of *T_g_* and *G′_rubbery_*_,_ is highest when irradiation occurs in ambient air, presumably because oxygen can react with irradiation-induced radicals, thus preventing the recombination of cut chains.

For thin samples and lower dose rates, the effect of oxygen on cross-linking and chain scission rates may be more pronounced than for the 4-mm-thick samples used in this study, where the processes might be oxygen diffusion-limited.

### 4.3. Effect of the Irradiation Temperature

When irradiating the CTD101K, MSUT, and CEA mix epoxy systems in liquid helium, the initial increase of *T_g_* and rubber modulus that occurs during ambient temperature irradiation is not observed, or it is very small. This indicates that cross-linking rates are strongly reduced when irradiating in liquid helium, presumably because, due to the limited molecular movement, cross-linking processes are largely suppressed.

For the epoxy resin systems CTD101K, MSUT, CEA mix, Mix61, and MY750, the DMA results show that chain scission at 4.2 K occurs at a lower rate than at ambient temperature, confirming previous results showing a strong reduction of chain scission rates for irradiations in liquid nitrogen [29] and liquid helium [30]. For the Araldite F epoxy system, the aging rate appears to be similar for irradiations in air, inert gas, and liquid helium.

### 4.4. Comparison of the Radiation Hardness of the Different Epoxy Resin Systems

The MSUT and CDT101K irradiation-induced aging rates derived from the DMA data indicate that these two resin systems are the most irradiation-resistant in this study. MSUT exhibits a particularly low chain scission rate up to 20 MGy, which is the highest dose of the present study. Irradiation of CTD101K and MSUT to higher doses and testing of the irradiated samples are ongoing.

The effect of ambient air irradiation on MY750 and Mix61 is comparatively higher [8], and these epoxy resins are not recommended for applications where high doses will be absorbed. These results confirm previous results where neutron-induced outgassing rates of MSUT and CTD101K are lower than those of Mix 61 and MY750 [8].

The results of the present study confirm that the radiation hardness is only partly determined by the epoxy resin and that the choice of additives like hardeners is crucial for application in an irradiation environment [31,32,33].

As an example, the epoxy systems MSUT, Araldite F, and MY750 based on the same bisphenol A/epichlorohydrin resin exhibit strongly different aging behaviors. Similarly, the CTD101K and Mix61 epoxy systems are based on the same bisphenol A diglycidyl ether (the molecular mass of the liquid epoxy resin is 340.5 g/mol) but have strongly different radiation hardnesses. This indicates that the radiation hardness is strongly influenced by the hardener and/or other additions like a flexibilizer or accelerator.

The strong influence of the hardener can be seen by a comparison of the radiation hardness of Araldite F epoxy resin in combination with different hardeners. In the present study, Araldite F was thermally cross-linked with the carboxylic anhydride hardener Aradur HY 905. This system is much more resistant to radiation than the previously studied Araldite F that was cured with a polyetheramine hardener (Jeffamine D400).

The Araldite F system studied here contains the polyglycol flexibilizer DY 040. It was shown previously that when this flexibilizer is added to the CTD101K system, it does not strongly change the radiation hardness of the epoxy system.

### 4.5. Future Irradiation Needs

To predict the evolution of the functional properties of the insulation systems in superconducting magnets in an irradiation environment, the DMA measurements need to be complemented by mechanical, thermal, and dielectric tests of samples irradiated at cryogenic conditions. Such measurements require a much larger sample volume than the DMA measurements, and to ease destructive testing, samples should not be radioactive after irradiation.

A cryo-cooled sample holder installed in a gamma-ray source would allow one to irradiate superconducting magnet insulation systems under realistic conditions and to perform stress strain measurements and dielectric tests of such irradiated materials. Cryocooled irradiation set-ups have already been successfully used, for instance, for testing the radiation hardness of LHC bypass diodes at cryogenic temperatures in CERN’s CHARM irradiation facility [34]. The diodes were thermally connected to the cryocooler cold head inside the radiation facility, and the cold head was connected via 40-meter flexible lines to the helium compressor that was placed outside the irradiation bunker. Such a system could also be installed at gamma-ray facilities, provided that sensitive cryocooler constituents like rubber seals can be avoided or sufficiently shielded such that the set-up can resist the irradiation environment.

## Figures and Tables

**Table 1:** Comparison of the glass transition temperatures derived from G′ onset, G″ max, and tan δ max and the linear thermal expansion coefficient (α) in the temperature range 30–45 °C. * E’ onset at a heating rate of 4 K/min. Densities at 23 °C have been measured according to the standard [27].

Material	ρ (g/cm^3^)	*T_g_* (°C)			α (×10^−6^ K^−1^)
		G′ Onset	G″ Max	tan δ Max	
CTD101K	1.22	118	122	140	60
MSUT	1.23	88	91	107	60
CEA mix	1.27	100	107	113	56
Araldite F	1.22	91	93	105	55
MY750	1.15	49	51	55	62
Mix61	1.14	−38 *	n.m.	73	144

## Data Availability

Data are contained within the article.
